# Exploiting Differential Gene Expression to Discover Ionic and Osmotic-Associated Transcripts in the Halophyte Grass *Aeluropus littoralis*

**DOI:** 10.1186/s12575-019-0103-3

**Published:** 2019-07-15

**Authors:** Farzaneh Fatemi, Seyyed Hamidreza Hashemi-petroudi, Ghorbanali Nematzadeh, Hossein Askari, Mohammad Reza Abdollahi

**Affiliations:** 1 Department of Genetic Engineering and Molecular Biology, Genetic and Agricultural Biotechnology Institute of Tabarestan (GABIT), Sari Agricultural Sciences and Natural Resources University (SANRU), P.O. Box 578, Sari, Iran; 2 Department of Biotechnology, Faculty of New Technologies and Energy Engineering, Shahid Beheshti University, Tehran, Iran; 3 Department of Agronomy and Plant Breeding, Faculty of Agriculture, Bu-Ali Sina University, Hamedan, Iran

**Keywords:** Salinity, Ionic effects, Osmotic effects, *Aeluropus littoralis*, cDNA-AFLP, RT-qPCR

## Abstract

**Background:**

Salinity as a most significant environmental challenges affects the growth and productivity of plants worldwide. In this study, the ionic and iso-osmotic effects of salt stress were investigated in *Aeluropus littoralis* L., a halophyte grass species from Poaceae family, by cDNA-amplified fragment length polymorphism (cDNA-AFLP) technique. To dissect the two different effects (ionic and osmotic) exerted by salt stress, various ionic agents including 200 and 400 mM sodium chloride (NaCl), 200 and 400 mM potassium chloride (KCl) as well as 280 and 406 gl^− 1^ (− 0.9 and − 1.4 MPa) polyethylene glycol 6000 (PEG) as their iso-osmotic concentrations were applied.

**Results:**

Application of KCl and PEG significantly reduced the fresh weight (FW) of *A. littoralis* seedlings compared to control while NaCl treatment markedly enhanced the FW. At the transcriptome level, different observations of changes in gene expression have been made in response of *A. littoralis* to ionic and osmotic stresses. Out of 69 transcript derived fragments (TDFs), 42 TDFs belong to 9 different groups of genes involved in metabolism (11.6%), transcription (10.2%), ribosomal protein (8.7%), protein binding (8.7%) transporter (5.8%), translation (5.8%), signal transduction (4.3%), nucleosome assembly protein (2.9%) and catabolism (2.9%). The 44 and 28 percent of transcripts were expressed under ionic stress (NaCl-specific and KCl-specific) and osmotic stress (common with NaCl, KCl and PEG), respectively which indicating a greater response of plants to ionic stress than osmotic stress. Expression pattern of eight candidate TDFs including; *SYP81*, *CAND1*, *KATN*, *ISB1*, *SAMDC*, *GLY1*, *HAK18* and *ZF30* was evaluated by RT-qPCR at high salinity levels and recovery condition.

**Conclusion:**

Differential regulation of these TDFs was observed in root and shoot which confirm their role in salt stress tolerance and provide initial insights into the transcriptome of *A. littoralis*. Expression pattern of ionic and osmotic-related TDFs at *A. littoralis* can be taken as an indication of their functional relevance at different salt and drought stresses.

## Background

Crop production is adversely affected by various environmental stresses. Many biochemical, physiological and molecular changes occur when plants frequently exposure to different stress conditions. These changes are resultant of massive regulations in the profile of gene expression [1]. Drought and salt stress physiology overlaps and cross-talks with each other. Salt stress generates lower water potential in the zone, making it difficult for the plant to absorb water leading to dehydration of the cell and ultimately disruption of osmotic equilibrium. Therefore, the form of a physiological drought is taken in the plant under the salt stress [2]. Na^+^, K^+^, H^+^ and Ca^2+^ are the major ions involved in signal transduction. Restoring the osmotic balance of the cell, damage repair and control by the maintenance of cellular homeostasis, detoxification and signaling to coordinate cell function are mechanisms that plants use in response to salinity and drought stresses [3, 4].

Salt stress consists of two main components including osmotic effect and ionic effect [5, 6]. Osmotic effect decreased water absorption in the rhizosphere, while ionic effect results to imbalance or intercellular toxicity due to excess ions [7]. Many researchers have proved the existence of the ionic and osmotic components of salt stress. Singh et al. [8], showed that sodium chloride (NaCl) was more harmful for germination of pea when used as an iso-osmotic solution of polyethylene glycol 6000 (PEG). A study on K^+^ fluxes in the mesophyll of bean leaf under the mannitol and iso-osmotic NaCl treatments showed that different mechanisms are involved in the conception of ionic and osmotic components [9].

*Halophytes* are salt-tolerant plants growing exclusively in habitats with *high salinity* [10]*.* They can survive under the high salinities of NaCl. *Aeluropus littoralis* is a halophytic plant of Poaceae family. It is a monocotyledonous halophyte which usually grows in the regions with intermediate to high salinity [11, 12]. *A. littoralis* can tolerate up to 600 mM NaCl [13] or 800 mM NaCl [11]. *Aeluropus* species are potentially known as precious genetic resources due to accumulation of sodium and chloride ions in their over ground tissues and can improve our understanding about molecular mechanisms of salt and drought stress responses especially in cereals [14, 15]. Salinity stress increases the number and size of vacuole and also organelle density due to accumulating of Na^+^ and Cl^−^ fractions. Therefore, *A. littoralis* as a halophyte plant uses the same mechanism to overcome salt and drought stresses [11]. Many genes and biochemical–molecular mechanisms are involved in plant response to abiotic stress. Changes in gene expression profile are induced by a complex of signal transduction pathways that have not been determined clearly. Various genes respond to salinity and drought stress in some species and their functions have been predicted by alignment to known orthologous genes [5].

Serial analysis of gene expression (SAGE), representational difference analysis (RDA), differential display reverse transcription-polymerase chain reaction (DD-RT-PCR), suppression subtractive hybridization (SSH), c-DNA microarray and cDNA-amplified fragment length polymorphism (cDNA-AFLP) are techniques that currently are available for transcriptome analysis. Among these techniques, cDNA-AFLP as a transcriptome-wide screening tool [16] is an extremely efficient and less labor-intensive mRNA fingerprinting method for gene discovery [17] without any prerequisite knowledge about sequences [18]. This technique gives the possibility to identify rarely expressed sequence tags (ESTs) [19]. So, it provides rapid and multiple comparisons of the plant response to different stress durations and intensities [20].

In this research, the strategy is based on the discrimination of the whole salt stress effects into the ionic effect and osmotic effect. For this purpose, PEG was used as the non-ionic or iso-osmotic solution. We report the candidate genes that were differentially expressed in the roots under the NaCl, potassium chloride (KCl) and PEG treatments in *A. littoralis* using cDNA-AFLP method. The darkness condition was considered to omit the photosynthesis related genes due to its complexity and increasing the chance of identifying allocated genes to ionic and osmotic stresses. We also established a collection of stress-responsive ESTs in *A. littoralis* in NCBI GenBank and their possible functions and presumed biological implications were discussed based on the homology searches. This can help to identify salt and drought-inducible candidate genes in this halophyte plant for subsequent studies especially in the field of novel gene transfer.

## Results

### Effect of Ionic and Osmotic Stresses on Plant Fresh Weight

**Fig. 1:**
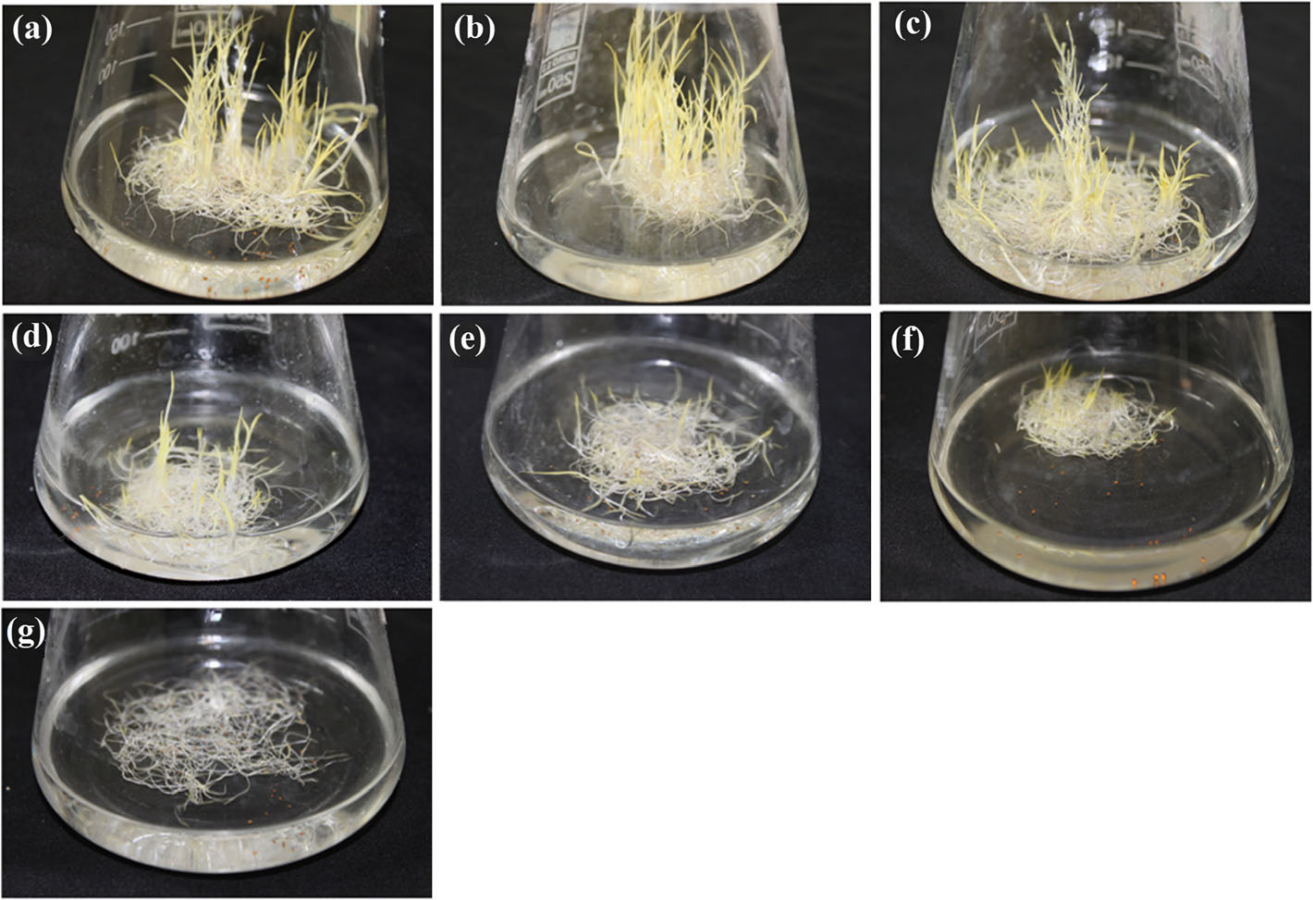
The effect of ionic and osmotic stresses on FW of *A.littoralis* in liquid MS culture medium. (**a**) Control culture medium, (**b** and **c**) culture media containing 200 and 400 mM NaCl, respectively, (**d** and **e**) culture media containing 200 and 400 mM KCl, respectively, (**f** and **g**), culture media supplemented with PEG 6000 preparing − 0.9 and − 1.4 MPa osmotic pressures, respectively

**Fig. 2:**
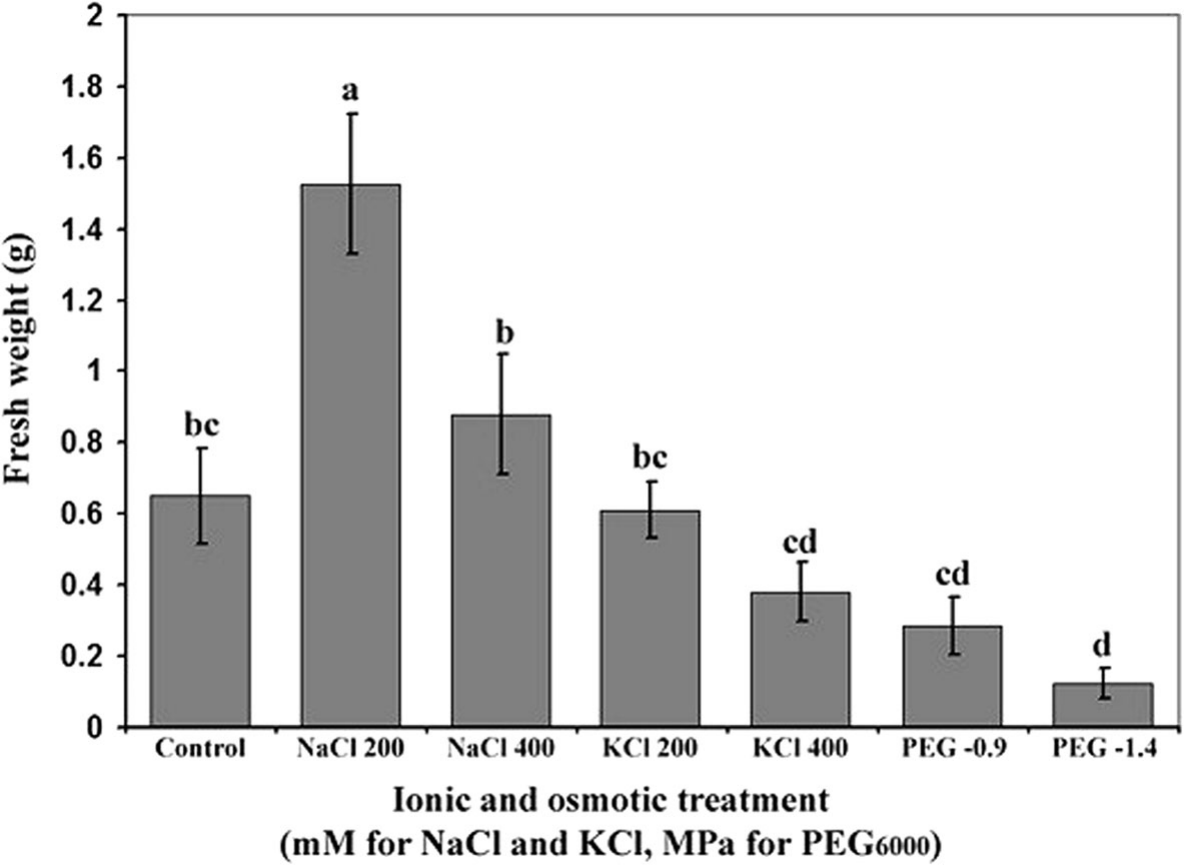
Effect of different ionic and osmotic treatments on FW of *Aeluropus littoralis* seedlings**.** The osmotic pressures, − 0.9 and − 1.4 MPa, prepared by 280 gl^− 1^ and 407 gl^− 1^ PEG 6000 solutions are iso-osmotic concentrations of 200 and 400 mM NaCl and KCl, respectively. The letters showed significantly difference at the 5% level according to Duncan’s multiple test. Significant differences between two bars marked with different letters

The FW of treated *A.littoralis* seedlings was evaluated in response to ionic and osmotic stresses. Both ionic and osmotic stresses induced by various levels of KCl and PEG significantly reduced the plant FW weight compared to control (Fig. 1 and Fig. 2) while use of 200 and 400 mM NaCl in liquid MS culture medium resulted in significant enhancement of FW compared to other treatments (Fig. 2). The reduction in plant FW was greater under PEG induced osmotic stress than stress induced by KCl treatments at their iso-osmotic concentrations (Fig. 2). Also, use of 406 gl^− 1^ PEG (400 mM or − 1.4 MPa) in culture medium resulted in more reduction in FW compared to 280 gl^− 1^ PEG (200 Mm or − 0.9 MPa) (Fig. 2).

### Identification of Ionic and Osmotic Stress-Induced Transcripts

**Fig. 3:**
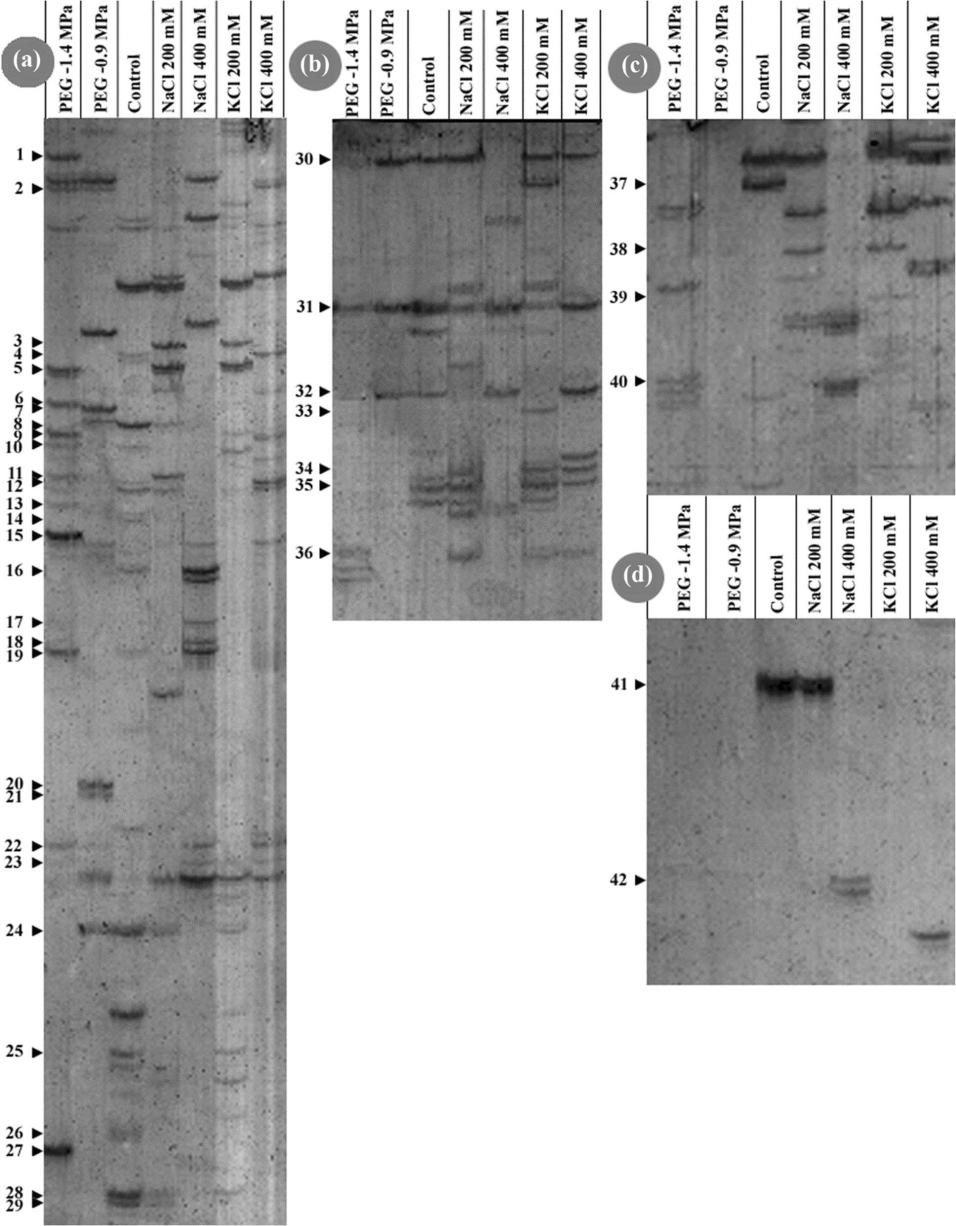
A representative picture of a silver-stained cDNA-AFLP gel showing the differential expression of the genes under different components of salt stress in *Aeluropus littoralis*. (**a**), (**b**), (**c**) and (**d**) are different sections of the main gel. Small black arrows show 42 TDFs described in Table 1

The cDNA-AFLP technique was used to isolate ionic and osmotic-responsive genes from *A. littoralis.* Noticeable differences were observed in gene expression profile between ionic and osmotic effects of salt stress during root development. Finally, 69 readable sequences were determined as the ionic and osmotic responsive genes which shared homology with genes encoding known, unknown, hypothetical proteins. Approximately 60.9% of the TDFs were shared identity to reported sequences in database. Some of the sequences showed homology to TDFs or genomic sequences of *Oryza sativa*, *Zea mays* and *Arabidopsis thaliana*, but 8.7% of the TDFs did not show any significant similarity to nucleotide or amino acid sequences in the GenBank and classified as no significant matches. TDFs with known functions are listed in Table 1 and Fig. 3.

**Table 1:** List of TDFs induced in cDNA-AFLP by KCl, NaCl and PEG treatments in roots of *Aeluropus littoralis.* TDFs were induced by 200 mM NaCl (N1), 400 mM NaCl (N2), 200 mM KCl (K1), 400 mM KCl (K2), − 0.9 MPa/280 gl-1 PEG (P1) and − 1.4 MPa/406 gl-1PEG (P2) in root. Sequences were compared to sequences in the GenBank database using the BLAST program. The E-value show the homology between the aligned sequences

TDF	Accession no.	Treatments	Length (bp)	Homology to gene	
				Name; accession number	E-value
1	JZ191042	P_2_	365	DUF21 domain-containing protein (*Brachypodium distachyon*); XM_003568464	9e-33
2	JZ191043	P_2,_ P_1,_ K_2_	350	Genomic DNA, chromosome 4, BAC clone: OSIGBa0158F13 (*Oryza sativa*); CR855151.1	4.0
3	JZ191047	N1, K1	312	Auxin response factor 3-like (*Glycine max*); XM_003529306.1	0.88
4	JZ191048	C, K2	284	Syntaxin 81 (*Zea mays*); EU963152	6e-46
5	JZ191088	P2, N1, K1	307	40S ribosomal protein S3 (*Glycine max*); XM_003548212	8e-55
6	JZ191049	P2	265	Aspartic proteinase (*Oryza sativa*); BAA02242.1	1.4
7	JZ191050	P1	261	Chloroplast envelope membrane protein (*Staurastrum punctulatum*); YP_636444	3.5
8	JZ191051	P1, C, N1	260	Voltage-dependent anion-selective channel protein 4 (*Oryza sativa*); Q0JJV1.3	3e-29
9	JZ191052	P2, K1	260	Myb-like DNA-binding domain (*Zea mays*); NM_001157897.1	0.59
10	JZ191054	P2, C	259	Myb-like DNA-binding domain (*Zea mays*); NM_001157897.1	2.8
11	JZ191055	P2, N1	247	G-box binding protein (*Oryza sativa*); EU847024.1	0.15
12	JZ191056	C, K2	259	40S ribosomal protein S12 (*Zea mays*); EU957837.1	2e-42
13	JZ191092	P2	260	Importin subunit beta-1-like (*Oryza sativa*); XP_015619891.1	8e-44
14	JZ191093	C	236	Nucleolin 2 (*Zea mays*); ONM05985.1	3e-08
15	JZ191094	P2	231	Putative glyoxalase I (*Oryza sativa*); BAD28547.1	0.008
16	JZ191096	C, N2	384	Serine/threonine-protein kinase SIS8 (*Brachypodium distachyon*); XM_010235744.3	4e-13
17	JZ191098	N2	212	Golgin candidate 4-like (*Brachypodium distachyon*); XM_003574367.1	4e-13
18	JZ191099	N2	207	Calcineurin B-like-interacting protein kinase (*Hordeum brevisubulatum*); JX679077.1	9e-17
19	JZ191100	P2, C, N2	237	Potassium transporter (HAK18) (*Brachypodium distachyon*); XM_010241173.1	7e-13
20	JZ191101	P1	180	Zinc finger CCCH domain-containing protein (*Oryza sativa*); XP_015632054.1	8e-20
21	JZ191102	P1	180	Zinc finger CCCH domain-containing protein 24 (*Oryza sativa*); Q10EL1.1	6e-19
22	JZ191103	P2, P1, N2, K2	170	Ubiquitin-related modifier (*Zea mays*); NM_001149704.1	3e-37
23	JZ191104	P2, N2, K1, K2	170	AP2/EREBP transcription factor ERF-1 (*Gossypium hirsutum*); AY779339.1	0.034
24	JZ191057	P1, C, N1, K1	159	Cullin-associated nedd8-dissociated protein1 (*Oryza sativa*); EF575856.1	9e-16
25	JZ191058	C, K1	136	S-adenosylmethionine decarboxylase (*Oryza sativa*); JN944362.1	5e-20
26	JZ191060	C	142	Metal-nicotianamine transporter (*Glycine max*); XM_003548246.1	2.6
27	JZ191061	P2	133	Zinc finger CCCH domain-containing protein (*Oryza sativa*); XM_003518517.1	6.7
28	JZ191062	C, N1, K1	126	Nucleotide binding site leucine-rich repeat (*Pyrus sinkiangensis*); ACJ05259	6.8
29	JZ191063	C, N1, K1	142	DNA glycosylase/lyase 701 (*Oryza sativa*); FJ536320	7.8
30	JZ191081	P1, C, N1,K1, K2	523	RNA binding protein (*Oryza sativa*); AAP85377.1	1e-37
32	JZ191070	P2, P1, C, N2, K2	391	60S ribosomal protein L38 (*Zea mays*); NP_001152328.1	1e-41
33	JZ191083	P1, K1	362	Cyclint 2-like protein (*Oryza sativa*); XP_015627068.1	2e-15
34	JZ191072	N1, K1, K2	361	Nucleosome assembly protein (*Brachypodium distachyon*); XP_003568000.1	0.44
35	JZ191073	C, N1, K1, K2	357	*Beta*-galactosidase (*Bathycoccus prasinos*); CCO19627.1	5e-05
36	JZ191075	P2, N1, K1, K2	329	Eukaryotic translation initiation factor p28 (*Zea mays*); NP_001104917.1	2e-65
37	JZ191064	C	566	Katanin p80 WD40 (*Brachypodium distachyon*); XP_003579480	1e-51
38	JZ191065	N1, K1	234	Dehydrin Xero 2-like (*Brassica rapa*); XM_013864438.2	7.9
39	JZ191066	K1	133	Glutamate decarboxylase-like (*Vitis vinifera*); XM_002263045.2	6.7
40	JZ191068	P2	403	ADP-glucose pyrophosphorylase (*Zea mays*); HM749416.1	0.005
41	JZ191107	C, N1	179	NADH dehydrogenase subunit J (*Passiflora incarnate*); KT721860.1	2.5
42	JZ191108	N2	152	Phytochrome C (*Cenchrus americanus*); JQ270557.1	0.027

### Classification of Expression Patterns

**Fig. 4:**
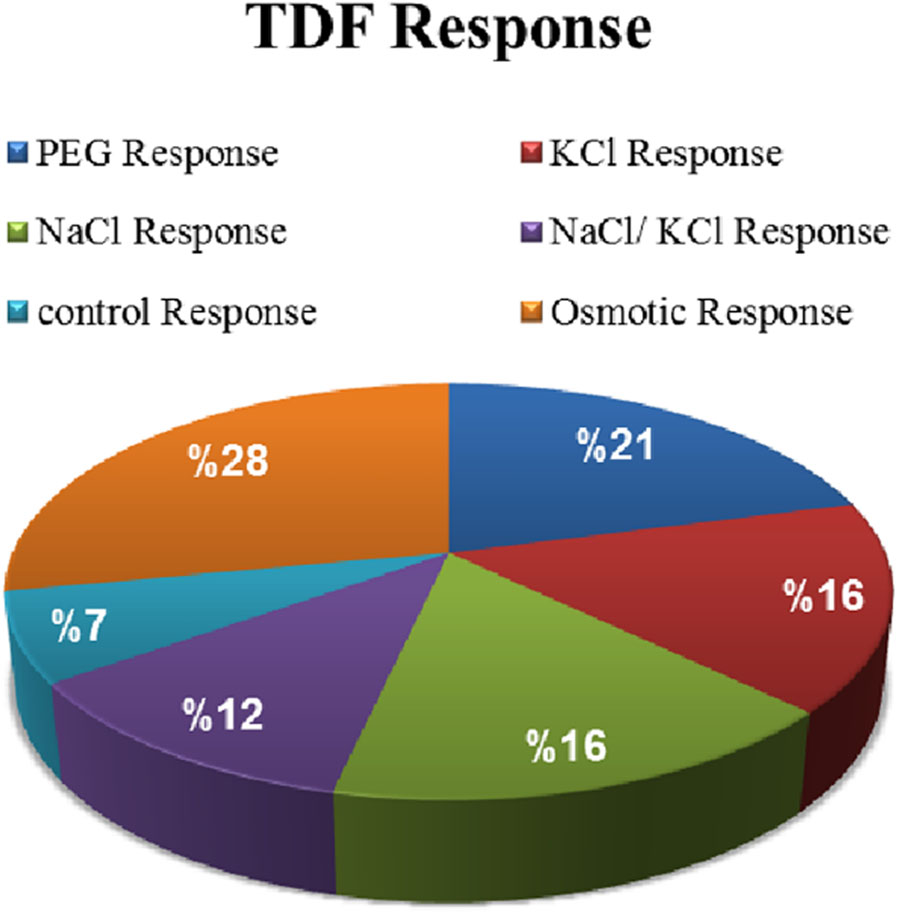
Expression patterns categories of *Aeluropus littoralis* roots in response to ionic and osmotic agents. The TDFs were classified into 6 groups based on presence/absence of bands

Different gene expression patterns were observed in the response of *A. littoralis* to ionic and osmotic stresses based on their presence/absence (qualitative variants). The TDFs classified into 6 categories. NaCl (specific to NaCl treatment), KCl (specific to KCl treatment), PEG (specific to PEG treatment), KCl/NaCl (common with ionic NaCl and KCl treatments) and KCl, NaCl/PEG (common with all treatments). TDFs in the NaCl and KCl categories, display the genes that are directly related to the ionic effect, which were not expressed in PEG treatment. Similarly, the KCl, NaCl/PEG category reflects the osmotic effect, and the PEG category should represent specific effects of PEG chemicals rather than the osmotic effect. Such extra effects of PEG have been reported previously [21]. In the following classification (Fig. 4), a majority of the TDFs (44%) fell into the group that represented ionic response (NaCl 16%, KCL 16% and NaCl/KCl response 12%) whereas 28% of TDFs showed osmotic response. The rest of the TDFs belonged to PEG and control response (21 and 7% respectively) (Fig. 4).

### Functional Determination of TDFs

**Fig. 5:**
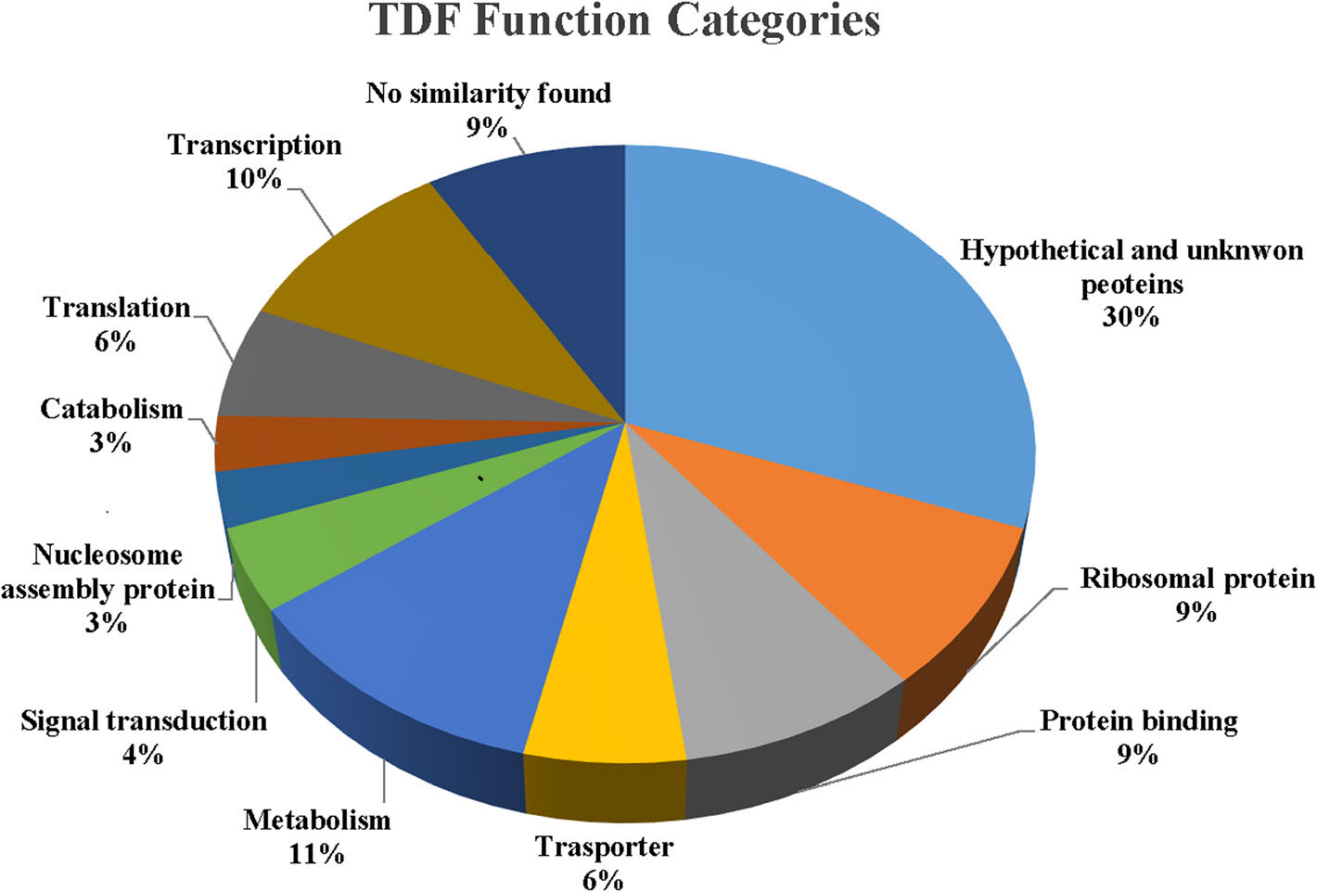
Functional percentage distribution of TDFs in *Aeluropus littoralis* based on gene functions obtained from Gene Ontology [22]

The sequence comparison of 69 readable TDFs against the database revealed that most of them had homology to genes with known functions (Fig. 5), whereas, 30.4% (21 TDFs) of TDFs belonged to either hypothetical or unknown function proteins, and 8.7% (6 TDFs) of TDFs showed no significant matches. To obtain a better insight into the identity and possible functional of these stress-induced TDFs, the functional categories of TDFs were assigned based on gene ontology. The transcripts were grouped into 9 functional categories related to biological processes. The vast majority of annotations was involved in metabolism (11.6% - 8 TDFs), transcription (10.2% - 7 TDFs), ribosomal protein (8.7% - 6 TDFs) and protein binding (8.7% - 6 TDFs) (Fig. 5). Genes encoding proteins involved in transporter (5.8% - 4 TDFs), translation (5.8% - 4 TDFs), signal transduction (4.3% - 3 TDFs), nucleosome assembly protein (2.9% - 2 TDFs) and catabolism (2.9% - 2 TDF) formed the second largest groups.

### Differentially Expressed TDFs in Response to Ionic and Osmotic Treatments

The cDNA-AFLP analysis showed that the root-specific TDFs were highly expressed in ionic and osmotic stresses. Among 69 deposited TDFs to the dbEST database, 42 TDFs are presented in Table 1 and Fig. 3 with the GenBank accession numbers. The other 27 sequences were related to hypothetical/unknown protein and no similarity match in GenBank. We found that ionic and osmotic related TDFs was numerous in *A. littoralis*, especially the transcripts involved in metabolites and energy which should be considered to find out the mechanism of salt tolerance. Several TDFs like ribosomal proteins (Table 1 and Fig. 3, No 5, 12 and 32: JZ191088, JZ191056 and JZ191070), auxin response factor (Table 1 and Fig. 3, No 3: JZ191047), potassium transporter (Table 1 and Fig. 3, No 16 and 19: JZ191096 and JZ191100), NADH dehydrogenase (Table 1 and Fig. 3, No 41: JZ191107), S-adenosylmethionine decarboxylase (Table 1 and Fig. 3, No 25: JZ191058) and syntaxin (Table 1 and Fig. 3, No 4: JZ191048) are strong candidates that are specific to the ionic effects (NaCl and KCl). Some other TDFs such as transcription factors (Table 1 and Fig. 3, No 23: JZ191104), RNA binding protein (Table 1 and Fig. 3, No 30: JZ191081), cyclin (Table 1 and Fig. 3, No 33: JZ191083), translation initiation factor (Table 1 and Fig. 3, No 36: JZ191075), zinc finger CCCH domain (Table 1 and Fig. 3, No 20, 21 and 27: JZ191101, JZ191102 and JZ191061), ubiquitin (Table 1 and Fig. 3, No 22: JZ191103), are expressed in response to osmotic stress (common with NaCl, KCl and PEG) (Table 1 and Fig. 3).

### Expression Patterns of the cDNA Fragments

**Fig. 6:**
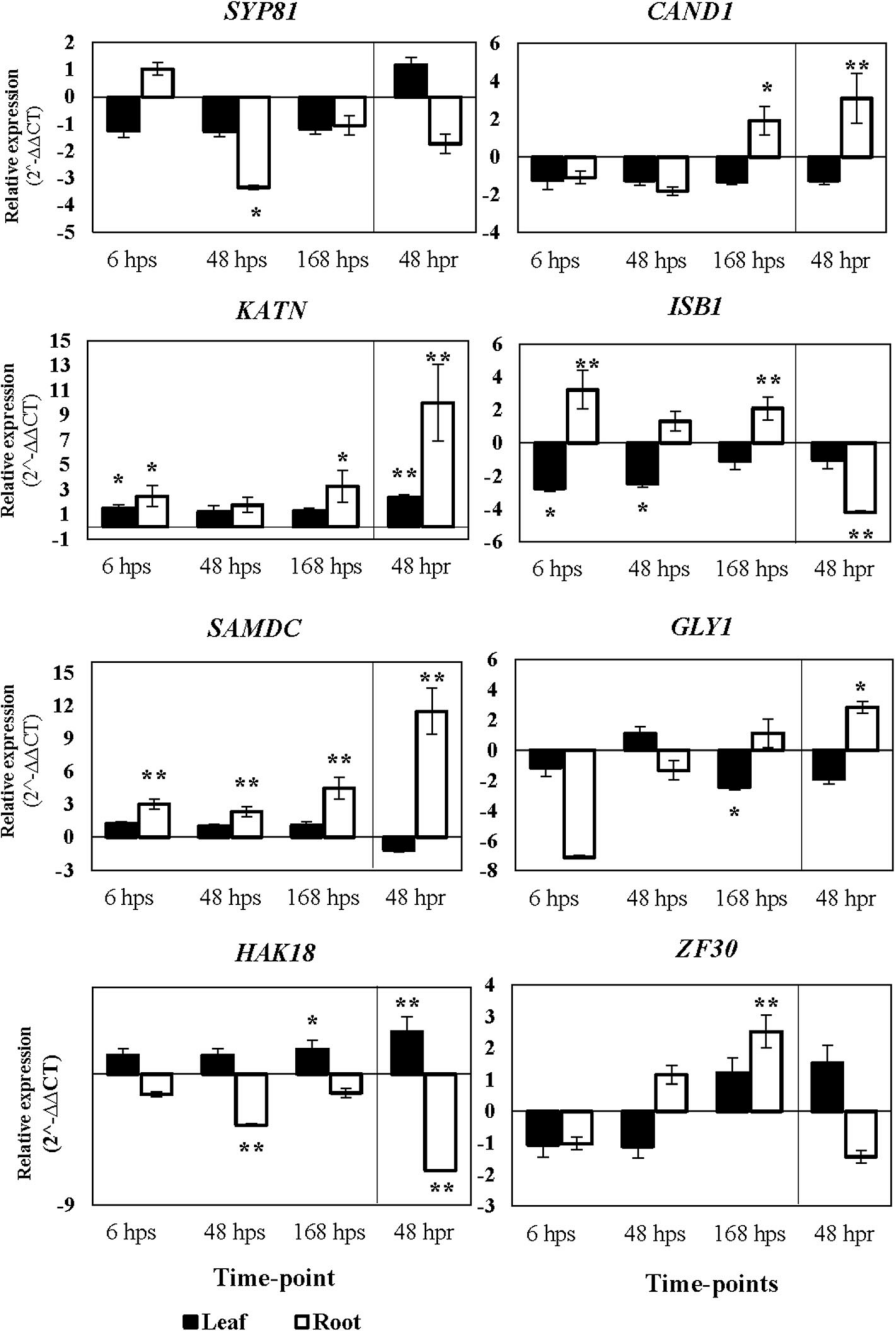
Trend of regulated genes during different time-point of salt stress and recovery condition. *SYP81*, Syntaxin of plants 81; *CAND1*, Cullin-associated and neddylation-dissociated; *KATN*, Katanin p80 WD40; *ISB1*, Importin subunit beta-1; *SAMDC*, S-adenosylmethionine decarboxylase; *GLY1*, Glyoxalase I; *HAK18*, High-affinity potassium transporter; *ZF30*, Zinc finger CCCH domain-containing protein 30. hps and hpr are the abbreviated of hours post stress and hours post recovery, respectively. A single asterisk (*) and double asterisks (**) represent significant difference from the control (0 hps) (*P* < 0.05, n = 3) and very significant difference from the control (0 hps) (*P* < 0.01, *n* = 3), respectively. The relative expression based on 2^-∆∆CT is represented in the y axis and the time-point is represented in the χ axis

**Fig. 7:**
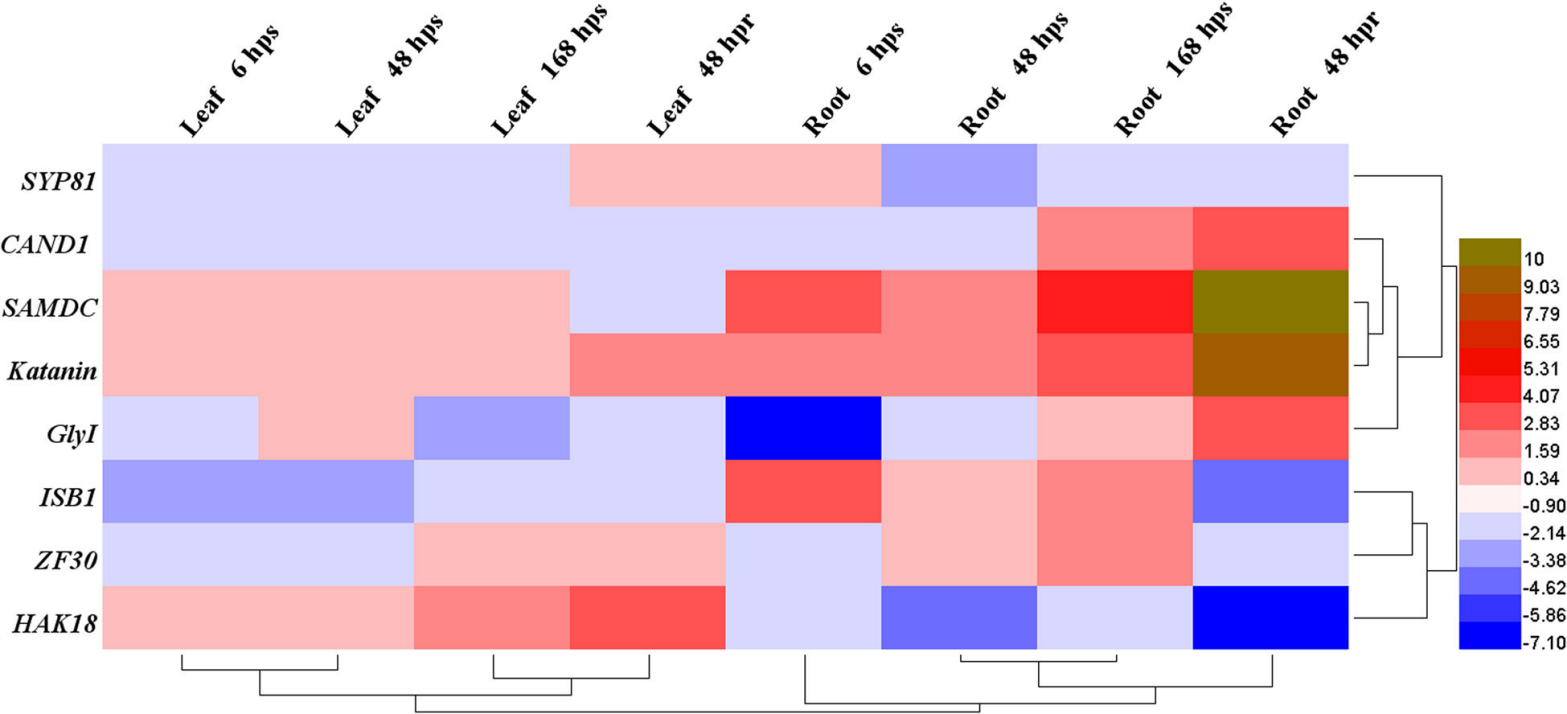
Heat-map showing RT–qPCR expression of eight TDFs against salt stress and recovery condition in leaf and root tissues of *A. littoralis*

The gene expression patterns of eight TDFs including *SYP81*, Syntaxin of plants 81; *CAND1*, Cullin-associated and neddylation-dissociated; *KATN*, Katanin p80 WD40; *ISB1*, Importin subunit beta-1; *SAMDC*, S-adenosylmethionine decarboxylase; *GLY1*, Glyoxalase I; *HAK18*, High-affinity potassium transporter; *ZF30*, Zinc finger CCCH domain-containing protein 30 were individually assessed in 600 mM of NaCl stress and recovery condition by using RT-qPCR due to their important role in ionic and osmotic stresses. It has been reported that at high salinity levels, the ionic effect dominates or equals to the osmotic effect [23]. Values were determined to statistically significant fold changes with 95% confidence (*P* = 0.05). Genes with fold changes are indicated in Fig. 6. In general, all the genes had significant difference relative to control. At time-point 6 hps, the expression level of *SYP81, KATN*, *SAMDC* and *ISB1* were higher while *CAND1*, *GLY1*, *HAK18* and *ZF30* were down-regulated in root (α < 0.05). All 8 genes followed the same pattern in 48 hps and 168 hps time-points except *CAND1* and *GLY* (Fig. 6). Under recovery conditions at time-point 48 hpr, the expression level of *CAND1, KATN, GLY1* and *SAMDC* increased relative to control while, the genes of *SYP81, ISB1, HAK18* and *ZF30* showed down-regulation in mRNA level. Similar to root analysis, expression values of eight genes across three time-points of leaf samples were also examined. The genes *SYP81, KATN*, *SAMDC*, *ISB1, CAND1, HAK18* and *ZF30* followed the same pattern in 6 hps and 48 hps time-points except *GLY1.* In 168 hps time-point, *KATN*, *SAMDC*, *HAK18* and *ZF30* were up-regulated whereas *SYP81, ISB1, GLY1* and *CAND1* were significantly down-regulated. In 48 hpr time-point, up-regulation of *SYP81, KATN*, *HAK18* and *ZF30* were observed among all analyzed genes. The heat-map generated from RT-qPCR expression data represented the differential transcript abundance of the eight candidate TDFs in salt stress and recovery condition in both leaf and root tissues (Fig. 7). The largest gene expression values are displayed in light green color while the dark blue showed smallest values. Furthermore, it has shown that the TDFs under investigations cluster together based on their induction at different time-points (Fig. 7). Assigning subcellular localization to a protein is also an important step towards elucidating molecular function and its interaction partners. For predicting protein subcellular localization of each TDF, the protein sequence of each TDF homologues in *S. italica* was analyzed by Plant-PLoc program (Table 2).

**Table 2:** The list of candidate TDFs and their gene specific primer sequences that were used for RT-qPCR. Prediction of subcellular localization was done base on their gene homologues in *Setaria italica*

Gene symbol	Name	Function	Gene homologues in *Setaria italica*	Predicted subcellular location	Primer sequence	Amp. size
*SYP81*	Syntaxin of plants 81	Vesicle trafficking protein that functions in the secretory pathway.	XP_004976323	Nucleus	CAGCATGGCGTGGCTCTTAT AGCATCTTGAAAGCGCATGG	90
*CAND1*	Cullin-associated and neddylation-dissociated	Key assembly factor of SCF (SKP1-CUL1-F-box protein) E3 ubiquitin ligase complexes that promotes the exchange of the substrate-recognition F-box subunit in SCF complexes	AT2G02560 cell-to-cell mobile RNA XP_004951789.1	Chloroplast	TGGCAGTGACTACAGCATACGG ACTGCGCACAGAGCGGTACT	91
*SAMDC*	S-adenosylmethionine decarboxylase	Essential for polyamine homeostasis, and normal plant embryogenesis, growth and development.	XP_004953064.1	Cytoplasm	CCATCCATGGTCCTGCTTTC GGGTTGAAGCCCATGACCTC	81
*Katanin*	Katanin p80 WD40	Microtubule severing	XP_012700331.1	Chloroplast	TGATCCCTCCCTTCCCAGTT CCTGAGCGAATGCGTAAACC	98
*ISB1*	Importin subunit beta-1	Protein transporter activity	XP_004962709.1	Cytoplasm	GCTCCAGCCAAATGTCAAGC GGTCTTGGTCAACAGCTTCAGG	86
*GlyI*	Glyoxalase I	Carbohydrate metabolic process	XP_004952236.1	Chloroplast	GTGGCATGGACTTGCTACGG CCGTGGCATCACAGAGGATT	92
*HAK18*	High-affinity potassium transporter	Potassium ion transmembrane transporter activity	XP_004956156.1	Plasma membrane	GGCCAGACATTTCAGACCACA AGCCCTGATGACCGTGTTTC	99
*ZF30*	Zinc finger CCCH domain-containing protein 24	Regulation of transcription	XP_004982091.1	Nucleus	GCTCTTGTTGGCTCCCCTCT TCACCATTTACGCCCCAATC	83
*RPS3*	40S ribosomal protein S3-like	Structural constituent of ribosome involved in RNA methylation, photorespiration, translation	XP_004972758.1	Chloroplast	ATTCACTGGCTGACCGGATG GTGCCAAGGGTTGTGAGGTC	107
*UBQ*	Ubiquitin-like protein	Biologically significant role in protein delivery to proteasomes and recruitment of proteasomes to transcription sites	XP_004957594.1	Chloroplast	CTTGGTCTGCTGTTGTCTTG CACGGTTCACTTATCCATCAC	200
*EF1A*	Elongation factor-1 alpha	Translation elongation factor activity	XP_004984833.1	Cytoplasm	TGCTGTCGGTGTCATCAA CTTCCATCAAACGCCTCATT	97
*U2SURP*	U2snRNP-associated SURP motif-containing protein-like	RNA binding, required for spliceosome assembly to participate in splicing	XP_004951689.1	Nucleus	CGTGGATGAGATTGAGAGGAA TGGAGGACTACGGCTTCTA	199
*GTF*	General transcription factor 3C polypeptide	Involved in RNA polymerase III-mediated transcription	XP_004975210.1	Nucleus	TTCCAAGTGGCCATCAGGTT AAAGGGCTTCCTGCCTCTTG	108

## Discussion

In the present study, the molecular response of *A. littoralis* seedlings to different ionic agents (KCl and NaCl) and osmotic agent PEG, as their iso-osmotic concentrations, were investigated to separate the ionic and osmotic effects of salinity. The plant FW was used in term of physiological growth index to evaluate toxicity in tolerant samples exposed to ionic and osmotic agents. Differentially expressed cDNA fragments from whole plant were assessed in etiolated samples to increase chance of isolating non photosynthesis-related genes. Also, expression pattern of eight candidate TDFs was evaluated at high salinity level to validate and confirm their functional relevance for regulating ion homeostasis and osmotic tolerance in *A.littoralis*. Putative gene functions of ESTs were also classified as described by Zouari et al. [15].

The present results showed that iso-osmotic stresses developed by NaCl, KCl and PEG agents significantly reduced the plant FW of *A. littoralis*. Several previous studies compared the effects of different salts and osmotic stresses in different plant species. Different responses of plant FW to different ionic and non-ionic treatments at iso-osmotic concentrations indicated specific ionic and non-ionic effects [24]. Osmotic stress induced by PEG exhibited more reduction in FW in comparison to ionic stress (NaCl and KCl treatments) at iso-osmotic levels indicating the inhibitory effect of osmotic stress than ionic stress [24].

On the other hand, different growth responses to different concentrations of iso-osmotic salt solutions indicated specific ionic effects. Increasing the biomass production of the seedlings at 200 mM of NaCl treatment in compered to all treatment showed the halophyte nature of *A. littoralis* while reduction in FW at 400 mM of NaCl treatment indicated threshold of tolerance in this plant.

Unexpectedly, KCl caused a greater inhibition of plant FW than control especially at 400 mM (− 1.4 MPa) (Figs. 1 and 2) due to toxic effects of ionic elements and a noticeable inhibition imposed by osmotic effect especially at − 1.4 MPa PEG [25]. Decreasing of plant growth in response to KCl stress at both 200 and 400 mM, suggesting that 200 mM KCl could be considered as threshold of tolerance. The result showed that potassium chloride is more toxic than sodium chloride and K^+^ ion would affect plant growth more than Na^+^ ion supporting the results of the research on *Atriplex prostate* [26]. So the inhibitory effect of salt stress and osmotic stress on plant growth and survival follow the pattern; PEG >KCl > NaCl. These findings are consistent with the results obtained from other researchers [24].

cDNA-AFLP is a whole transcriptome-wide technique for isolating tissue-specific genes in a wide range of biological systems and especially for the large-scale analysis of gene expression in plants [17]. Unlike DNA-microarray technology, cDNA-AFLP does not address transcript abundance exactly but it is helpful for identifying the change of transcript abundance without comprehensive genomic information or prior sequence knowledge [27]. Here we used mRNA Capture Kit with one-tube method and the advantage of using streptavidin-coated PCR tubes in which, mRNA reverse transcription and cDNA restriction can be performed in a one-tube format efficiently and eliminate several time-consuming steps. Salinity consists of multiple components including ionic and osmotic effects and this is the important reasons for the complexity of salt tolerance. cDNA-AFLP indicates the differences and similarities in gene expression profile between the ionic effect and osmotic effect successfully. In this study, cDNA-AFLP was used to detect the differences in gene expression profile between ionic or osmotic effects of salt stress. Transcript-derived fragments with potentially relevant function in ionic and osmotic tolerance were identified based on the differential expression pattern. However, limited information was obtained on the sequences and gene expression in *A. littoralis.* So, it was not surprising that 27 of the 69 TDFs identified as hypothetical or unknown proteins (30.4%) and 6 of them showed no similarity match (8.7%) to the presented data on NCBI. The results detected by cDNA-AFLP confirmed that all the selected TDFs from JZ191042 to JZ191110 showed specific expression pattern. Similar results have been obtained from cDNA-AFLP analysis of salt-stressed soybean [7], such as, CCCH-type zinc finger protein, ubiquitin, glutamate, envelope membrane protein, anion-selective channel protein, putative protein kinase, etc.

In cDNA-AFLP analysis, TDFs specific to ionic effect were remarkably more abundant in the root (44%) than those specific to the osmotic effect (28%) (Fig. 3). This indicates the importance of roots in stress conception. Previous studies have also noted that roots play an important role for limiting ion accumulation in shoot [7]. In this research, TDFs of JZ191061, JZ191100, JZ191083 and JZ191048 were homologous to zinc finger CCCH domain, potassium transporter, cyclin, and syntaxin, respectively. These TDFs are candidates for regulating ion homeostasis and osmotic tolerance in *A. littoralis*. The RT-qPCR was done for these genes; *SYP81*, *CAND1*, *KATN*, *ISB1*, *SAMDC*, *GLY1*, *HAK18*, ZF30. The quantification of given transcripts was performed to determine the changes in the transcript levels under exogenous treatments. In the present study, we have focused on gaining insight on the differential expression of some ESTs in response to salt stress and recovery condition which were estimated by RT-qPCR both in root and leaf tissues. Different responses were found to ionic and osmotic treatments. Interestingly, expression of some genes was induced by salt stress while also significantly repressed by recovery condition.

The zinc finger CCCH domain plays a role during the osmotic stress. The TDF of JZ191061 was homologous to zinc finger CCCH domain-containing proteins from *Glycine max*, and found to be involved in different biological processes including regulatory, signal transduction, DNA and RNA binding, zinc ion binding, mRNA processing and various biotic and abiotic stress responses [28, 29]. Investigation of the *cis*-elements in the promoter regions of the maize CCCH genes which response to stress showed similarity to two types of *cis*-elements, such as the ABA-responsive element and dehydration-responsive element. So, it can be concluded that the CCCH genes contain ABRE or DRE as the drought stress-responsive genes in their promoter sequences [30]. The zinc finger protein encoding transcription factors was previously reported as stress-inducible genes by Zouari et al. [12].

The cDNA fragment JZ191048 was homologous to syntaxin 81 in *Brachypodium distachyon*. Physiological information showed that plant response to salinity and drought stresses is controlled by expression of a set of particular genes. The proteins derived from these genes are involved in plant protection against the adverse effects of stress and minimize the damage caused by them [31, 32]. The studies confirmed the key roles of syntaxin in mediating the vesicle trafficking between the plasma membrane and the Golgi [33]. They regulate transport and activity of both aquaporins and plasma membrane K^+^ channels, which involved in the regulation of water homeostasis in the cell as a result playing an important role in osmotic adjustment during cell expansion and environmental stresses [34].

The TDF JZ191083 exhibited similar pattern to cyclin proteins which regulate cell cycle and cell division but their exact function in abiotic stress are largely unknown. It is obvious that undesirable conditions prevent root growth due to regulation of cell division and cell cycle [35]. Cellular studies showed that growth is as a result of produced cells in the meristem and the final length of the cells at the end of the growth zone [36]. So, cell division partially specifies organ elongation rate by controlling the dividing cells number and also the average cell cycle duration. From this viewpoint, salt stress decreases the rate of root elongation by reducing the final cells size and number of dividing cells resulted in shortening the size of meristems [37]. Salt stress stimulates root cells to elongate closer to the root tip rapidly, resulting in reduced meristem size and stopped cells division at a smaller size [38]. Compartmentalization of sodium and chloride ions in vacuole is one of the major adaptive responses of plants to decrease the toxic effect in the cytoplasm [39].

Moreover, the significant number of genes related to photosynthetic metabolism is down- or up-regulated in response to drought and salt [40]. It has been proved that the high need for osmotic adjustment and the need for reducing photosynthetic activity caused the changes in ATP amounts which is a response to the stress in shoots of *A. littoralis* [41]. Therefore, it can be concluded that the high capacity of ATP is used to provide energy for tolerance-related strategies in *A. littoralis*, which is a highly energy requiring process [40]. Based on these results, the seedlings were grown in a darkness condition to omit the impact of photosynthesis-related genes due to its complexity and increasing the chance of identifying allocated genes to salinity and drought stresses. Our results emphasize the importance of multiple effects of salt stress taking into consideration distinguishing between the ionic and osmotic effects by cDNA-AFLP method. Since *A. littoralis* is a member of Poaceae family, understanding the mechanism of salt tolerance and detecting the key genes involved in salt tolerance would be very helpful for breeding and genetic engineering of salt tolerant varieties in other members of the grass family including maize, wheat, barley, and rice. Our classification of TDFs based on their specific response to ionic and osmotic stresses should facilitate the functional analysis of stress-responsive genes.

## Conclusion

In this study, we identified 69 stress-induced genes in *A. littoralis*. The detailed study of 42 genes expression under several treatments with eliminating the impact of photosynthesis indicated that most of the dedicated transcripts were expressed under ionic stress than osmotic stress which in turn showing a greater response of *Aeluropus* roots to ionic stress. Several novel stress-responsive genes expressed in *A. littoralis* indicated special mechanisms of stress adaptability in this halophyte species. Determination the function, expression and translation of stress-inducible genes is necessary to understand the molecular mechanisms and to improve stress tolerance of crops by genetic engineering. The differential gene expression patterns observed in the physiological side suggest a high plant specificity in order to increase the chance of ESTs involved in stress tolerance. Our classification of TDFs based on the specific response to ionic and osmotic stresses could facilitate the functional analysis of salt-responsive genes in future studies of *A. littoralis*. The role of hypothetical and unknown TDFs in salt and drought response will need to be characterized further, and the information gleaned from such studies is expected to improve stress tolerance of crops by genetic engineering. This work will be continued by ectopic expression of candidate TDFs in either eukaryotic or prokaryotic expression system.

## Methods

### Plant Material Preparation

Seeds of *A. littoralis* were uncoated and sterilized for 1 min in 75% ethanol followed by 15 min in 2.5% commercial bleach, then rinsed three times with sterile distilled water. The decontaminated seeds were then placed in a flask containing 50 ml of full strength liquid MS medium [15] supplemented with 3% sucrose and vitamins. The pH of culture medium was adjusted to 5.8. Plantlets were grown in a growth chamber at 25 °C in the darkness condition and constant shaking (120 rpm) for seven days. These seven-day-old plantlets were subcultured and transferred to flasks containing 50 ml of liquid MS culture medium as described above. In order to study the ionic and osmotic effects, different concentrations of NaCl, KCl (200, 400 mM) and 280 and 406 gl^− 1^ PEG 6000, as their iso-osmotic concentrations (produced − 0.9, − 1.4 MPa), were added to subculture media according to Van’t Hoff equation [42]: (atm) π = icRT where, i is Van’t Hoff factor, which describes the number of particles per molecule dissolved; c refers to molarity of solution; R and T are universal gas constant (8.314472 LkPa/molK) and absolute temperature in Kelvin, respectively. This equation gives the osmotic pressure in Bar which should be converted to MPa.

After 14 days, the grown plants under the stress conditions and control were harvested. The whole plant fresh weight (FW) was measured, and then was quickly frozen in liquid nitrogen, and stored at − 80 °C for cDNA-AFLP analysis. The experiment was adjusted as a completely randomized design with five replications. The FW data were analyzed by analysis of variance (ANOVA). Significant differences between means were assessed by Duncan’s multiple range test (DMRT) at *p* < 0.05. SPSS 16 software was used to test the significant differences among levels of treatments.

### RNA Extraction and cDNA-AFLP Procedure

Total RNA from each stress treatment was isolated from about 100 mg of the frozen samples with the Trizol extraction kit (Invitrogen, USA) in three replications. Quality and quantity of isolated RNA were checked by agarose gel electrophoresis and spectrophotometer, respectively. The genomic DNA contamination was removed by DNase treatment (*DNase* I RNase-free, Thermo Scientific, USA). Root cells were lysed and mRNAs were isolated by capturing of poly(A+) RNA in streptavidin-coated tubes using a mRNA Capture kit (Roche, Switzerland). The first- and double-strand cDNA were synthesized by 5 μg of total RNA using a biotinylated oligo-dT primer in streptavidin-coated PCR tubes according to the manufacturer’s procedure. The restriction enzymes used for digestion of doubled strand cDNA were *Taq* I (Fermentase) and *Mse* I (Thermo Scientific, USA). The cDNA fragments released were purified and subsequently ligated to *Taq* I and *Mse* I. The pre-amplification reaction on ligation products was carried out using primers corresponding to *Taq* І (5′-GACGATGAGTCCTGACCGA-3′) and *Mse* I (5′-GACGATGAGTCCTGAG-3′) adapters. Twelve primer combinations were used for selective amplification; the primers were *Mse* І + 2: CC/CA/CT and *Taq* І + 2: GG/GC/AC/AT [18]. Selective amplification products (4 μl) were heat denatured and separated by electrophoresis in polyacrylamide gel (6%) containing 0.5X TBE. The gels were visualized by silver staining protocol and then were scanned by using imaging densitometer (GS-800, BioRad, USA).

### Isolation, Cloning and Sequencing of Transcript Derived Fragments (TDFs)

Discrimination of gene expression patterns to ionic and osmotic effects was assayed based on the presence/absence (qualitative variants) of the visualized bands on gel electrophoresis. Gel profiles were quantified using Quantity One gel image analysis software (version 4.4.1, Bio-RAD, USA) resulted in measurement of band intensities per lane for each time interval. The interested bands were marked and cut from the dried gels. The excised bands were eluted in 50 μl double distilled sterile water at 95 °C for 15 min then hydrated overnight at 4 °C. The eluted TDFs were reamplified by using the same set of selective primers under the PCR conditions as mentioned for AFLP. The PCR products were then checked on 0.5 x TBE 1.5% agarose gel. After confirming the size of the bands, the PCR products were ligated into the pTZ57R/T T/A cloning vector (InsTAclone PCR Cloning Kit, Thermo Scientific, USA), and were directly electro-transformed into DH5 competent *E.coli* cells. Colony PCR was done with M13 F/R primers and after confirming the size of the band, they were sent for sequencing (GATC, Germany).

### Bioinformatics Analysis

For TDFs in silico analysis, sequences of vectors and adaptors were trimmed off by using the VecScreen program on the NCBI website (www.ncbi.nlm.nih.gov/VecScreen). Translated sequences were analyzed for homology to publicly available GenBank non-redundant sequences databases (http.//www.ncbi.nlm.nih.gov/) using the BLASTX, BLASTN and TBLASX programs. Also, the Gene Ontology (http:// amigo1.geneontology.org/cgi-bin/amigo/go.cgi) and UniProt (http://www.uniprot.org/) database (https://www.arabidopsis.org) were used to investigate the molecular function of each TDF and its role in biological processes as well as their location in the cell. Finally, 69 TDFs were deposited in the GenBank dbEST database under BioSample number SAMN01924517; library number LIBEST_028119 (69 ESTs with accession numbers JZ191042– JZ191110). For predicting each candidate TDFs protein subcellular localization, the gene homologues of each candidate TDF was found in *Setaria italica* by BLASTX program. By using the obtained full sequence of protein, the subcellular localization was predicted by Plant-PLoc program [43].

### Reverse transcription–qPCR (RT-qPCR)

For RT-qPCR analysis, two-week-old seedlings were transferred to hydroponic culture containing Hoagland’s solution. The growth chamber conditions were 25 ± 2 C with 16 h light/8 h dark photoperiod at 600 μmol m^−2^s^-l^ photon flux density using cool-white fluorescent light. The two-month-old seedlings were stressed by 600 mM of sodium chloride (received 100 mM sodium chloride per two days). After reaching to 600 mM, leaf and root samples were collected at three time-points including 6 h post stress (hps), 48 hps and 168 hps. In order to plant recovery, the remained plants were transferred to a sodium chloride-free Hoagland’s solution, and then samples were collected 48 h post recovery (hpr). Control samples were also taken from unstressed plants at the initial of time-points. All samples were immediately frozen in liquid nitrogen and stored at − 70 °C for RT-qPCR analysis.

Total RNA was extracted using TRIzol reagent (Invitrogen Life Technologies, Karlsruhe, Germany) according to the manufacturer’s instructions. Equal quantity of RNA was used for cDNA synthesis. The cDNA was synthesized using the QuantiTect reverse transcription kit (Qiagen) according to the manufacturer’s instructions. The final cDNA reactions were diluted 1:5, and stored at − 20 °C. Gene-specific primers (Table 2) were designed according to the obtained sequences of the candidate TDFs (Table 1) using the Primer3.0 web resource (http://frodo.wi.mit.edu/cgi-bin/primer3/pri-mer3_www.cgi). The primer specificity was evaluated by melt curve analysis, and size of the amplicons was tested by end-point PCR on 3% agarose gels. For normalization of expression levels in *Aeluropus littoralis,* different sets of reference genes were selected for root and leaf samples according to the previous studies [13]. In this view, the three housekeeping genes (HKGs), namely *RPS3*, *EF1A* and *UBQ* were used as normalizer in root samples while two HKGs namely *U2SURP* and *GTF* were chosen for leaf samples. RT-qPCR was done by the Maxima SYBR Green/ROX qPCR Master Mix (Thermo Scientific) with two-step cycling in CFX96 real-time PCR instrument (Bio-Rad, USA) according to the company’s suggestions. Data acquisition was performed during the annealing/extension step. After amplification, all PCR reactions were subjected to a thermal melt with continuous fluorescence measurement from 55 °C to 95 °C for dissociation curve analysis. At least one non-template control (NTC) was used for each primer pair master mix. The PCR efficiency were approximated by the shape of the PCR amplification plot, and based on similar amplification plots of target and reference genes, the 2^-ΔΔCT method were used for calculation of relative gene expression ratio [44]. RT2 Profiler PCR Array Data Analysis software (SABiosystems, (QIAGEN, Germany) was used to construct Heat-map.

## Data Availability

The datasets measured and analyzed during the study are available from the corresponding authors upon reasonable request.

## Abbreviations

AFLP: Amplified fragment length polymorphism
*CAND1*: Cullin-associated and neddylation-dissociated
FW: Fresh weight
*GLY1*: Glyoxalase I
*HAK18*: High-affinity potassium transporter
*ISB1*: Importin subunit beta-1
*KATN*: Katanin p80 WD40
KCl: Potassium chloride
NaCl: Sodium chloride
PEG: Polyethylene glycol
RT-qPCR: Reverse transcription–qPCR
*SAMDC*: S-adenosylmethionine decarboxylase
*SYP81*: Syntaxin of plants 81
TDF: Transcript derived fragment
*ZF30*: Zinc finger CCCH domain-containing protein 30
